# From Molecular Phylogenetics to Quantum Chemistry: Discovering Enzyme Design Principles through Computation

**DOI:** 10.5936/csbj.201209018

**Published:** 2012-11-30

**Authors:** Troy Wymore, Charles L. Brooks

**Affiliations:** a Pittsburgh Supercomputing Center, 300 South Craig Street, Pittsburgh, PA 15213 USA; b University of Michigan, Department of Chemistry and Biophysics, 930 North University Avenue, Ann Arbor, MI 48109 USA

**Keywords:** Bioinformatics, Phylogenetics, Hybrid Quantum Chemical/Molecular Mechanical Methods, Enzyme Evolution, Structure-Function Relationships

## Introduction

Enzymes have obtained their amazing transformational capabilities through a colossal experiment in the optimization and diversification of protein structure-function relationships carried out over enormous stretches of time [1]. As such, it is critical to understand both the physicochemical properties of a biomolecular system and its history. Thus, the subject of this mini-review is on how both sequence-based bioinformatics and molecular dynamics (MD) simulations, particularly those using hybrid Quantum Chemical/Molecular Mechanical (QC/MM, the term QM/MM is also often used) potential energy functions, can be employed to discover the most critical amino acids relating to the enzyme's specific function as well as the atomic details of an enzymatic mechanism. We will highlight some results obtained from our own lab studying sesquiterpene synthases [2] and class D β-lactamases[3], two enzyme families known for their evolvability.

Comparative analysis of sequence and structural features of extant, mechanistically diverse, enzyme family members can result in the construction of powerful structure-function relationships and continues to dominate enzymatic studies today[4]. Yet, there remains an immense lack of knowledge at the atomic level on how enzymes evolve novel functions especially when residues outside the active site play a central role in this evolution. Some of the now classical studies of molecular adaptation, on topics such as insecticide resistance[5], color vision [6], antibiotic resistance [7–9], cofactor selectivity [10, 11], hormone receptor selectivity [12–14] as well as those from directed evolution [15], have uncovered a complex network of interactions often involving residues outside the active site. These studies have elucidated structure-function relationships that otherwise would have remained hidden with conventional analyses [1]. In addition, they have revealed stability-function trade-offs [16–18], promiscuity of ancestral proteins [19], and the profound concept of functional epistasis [20]. Functional epistasis is defined here as the phenotypic consequences of a mutation depending on the genetic sequence in which it occurs [21]. Epistasis restricts the evolutionary pathway to novel functions so that adaptive walks through sequence space must be acquired in a particular order and through a rugged functional landscape to avoid non-functional intermediates; an idea pioneered by Linus Pauling and Emile Zuckerkandl in a summary of their research [22] and by the evolutionary biologist John Maynard Smith in response to an attack on the concept of natural selection[23]. These studies obligate us to seek answers to broader, more fundamental questions [24] such as, “Does functional evolution proceed by a few mutations of large effect or by many mutations each of small effect?”; “Could alternative solutions to the same problem have evolved and if so how might they differ in sequence, structure and mechanism?”; “What role does epistasis have in structuring evolutionary trajectories?” and “Can we predict the course of genetic evolution?”[25]. The answers to these questions have deep implications for unlocking nature's fundamental design principles, understanding chemical allostery [26] and for developing totally new strategies for the rational design of molecules to control biochemical processes [27]. Achieving these remarkable insights into protein structure and function evolution will require a multi-disciplinary approach involving cutting-edge molecular biology, structural biology, bioinformatics and molecular modeling methods.

Enzymatic catalytic cycles often involve several chemical steps requiring the stabilization of multiple intermediate and transition states during catalysis. In principle, their active sites could be pre-organized to catalyze all of the chemical reactions involved (a principle championed by Arieh Warshel [28]) minimizing any reorganizational motions required to meet the demands of subsequent steps. This principle was demonstrated in a study of serine esterases [29]. However, if the active site reorganization needed to achieve catalysis of a subsequent step involves, for example, a conformational change, then evolution could have acted to 1) lower the energetic cost of reorganization and/or 2) evolve a novel function. In the latter case, protein fluctuations [30] play an important role in the mechanism and in understanding its evolution of novel function. In addition, the study of enzymes that show changes in catalytic activity upon mutation of residues distant from the active site presents a clear opportunity to better understand 1) allosteric principles [31, 32] to control, not just the binding of a substrate to the enzyme but also its reactivity with implications for the design of new natural products and the chemical rescue of disease proteins [33, 34], and 2) the vast neutral sequence space of proteins that endows them with two seemingly conflicting properties; robustness and evolvability [35].

## Molecular Phylogenetics

Molecular phylogenetics is now ubiquitous in most branches of biology [36] but can also be leveraged to make decisions on the type and locations of specific residues (those that are highly conserved or conserved only within an orthologous group) that would yield the most insight into structure-function relationships as well as aid in the design of species-specific inhibitors. This section describes the individual steps in carrying out a phylogenetic analysis and analyses based on a multiple sequence alignment (MSA). The initial step in a molecular phylogenetic analysis of an enzyme family is to gather enough protein sequences of sufficient variety in order to generate robust hypotheses from the subsequent analyses. For example, if a researcher would like to determine what residues are critical for specificity, then the data set must obviously contain several known paralagous members or sub-groups. Often, a BLAST [37] or PSI-BLAST [38] search is performed on the non-redundant protein database from NCBI using an appropriate query sequence to find orthologous and paralogous sequences. Alternatively, one could start from the iProClass database [39] that contains collections of sequences grouped according to protein function. Almost without exception, a researcher will then have to prune this dataset manually (to remove identical or nearly identical sequences and fragments) or with the aid of programs like CD-HIT [40]. This latter program is an excellent tool for clustering sequences and the subsequent selection of cluster representatives, often resulting in a diverse and more manageable set of protein sequences. The next step is to develop an accurate MSA for which several programs and online accessible interfaces are available. The most popular programs are T-Coffee [41], MUSCLE [42], ProbCons [43], and Clustal [44] and have been the subject of multiple reviews [45]. Yet, only in the relatively simple cases, do MSA programs get the entire alignment “correct” (as judged by comparison to a 3D structural alignment). Careful inspection and adjustment of the MSA should then be performed in some editor, like Genedoc [46] or Jalview [47] since even a highly conserved residue can be misaligned depending upon the degree of conservation in adjacent residues. Still, editing a large MSA can be a daunting task even with the help of an editor. Therefore, our group uses the Meme program [48] with the zoops model (zero or one motif per sequences) to search for the most conserved patterns or motifs ranging in length between six and 50 residues over the entire list of sequences. We have found that these parameters to MEME generally return motifs that are immensely useful for efficiently refining MSAs with the Genedoc editor (patterns/motifs are assigned a color which is highlighted over the MSA wherever the motif occurs, sb.nrbsc.org) as well as assisting in assigning sequences to groups (described in more detail below). The information content of a motif is determined by the conservation of residues along the motif, the length of the motif and the distribution of the motif residues in the submitted dataset [48]. In addition, a structural alignment of several 3-dimensional structures from the enzyme family can be performed with programs like STAMP within the MultiSeq module [49] contained within the VMD program [50] and used to assist in the MSA refinement. There are several very good multiple structure alignment programs [51] that all have similar performance when trying to align two proteins of similar length that share the same fold. The final MSA can then be designated “high-resolution” to distinguish it from the raw MSA program output.

The quality of a phylogenetic tree is highly dependent on the quality of the MSA and the regions included for tree construction [52, 53]. Therefore, the final MSA must be trimmed by deleting sections where the alignment is equivocal; primarily this trimming occurs at the N- and C-termini. Several automated programs, such as trimAl [54] and GBlocks [55], can be used to perform alignment trimming, though our manual trimming exercises have always resulted in trees with higher bootstrap values. The trimmed MSA file can then be used to create a distance-based phylogenetic tree or one based on maximum parsimony, Bayesian or maximum likelihood methods [36]. Once complete, several viewers are available for phylogenetic tree visualization [46]. Finally, organizing all of this sequence information and communicating the results to colleagues can still be problematic and tedious. Therefore, our group has developed a suite of utilities called HarvestSeq (sb.nrbsc.org) that will retrieve the functional characteristics for all sequences, order the MSA file based on the phylogenetic tree and perform other information gathering tasks and analyses.

The resulting phylogenetic tree can be used as a guide along with the gathered metadata on the sequences to partition the sequences into separate groups, typically those that cluster together with high bootstrap support. When the set of sequences is of a protein superfamily, the sequences cluster according to shared biochemical function. Sequences that have been incorrectly annotated are readily identified and with maximum likelihood or Bayesian methods, ancestral sequences can be inferred [56]. The construction of ancestral sequences has provided a wealth of information on how enzymes evolve new functions [56]. Other programs based on principle component analysis [57] and n-gram analysis [58] offer different but complimentary ways of grouping sequences. The MSA and a partitioning of sequences into defined groups can then serve as input to the GEnt program [3] that identifies amino acid residues characteristic of sub-groups within a set of orthologous proteins or characteristic of individual protein families within a collection of paralogous proteins. These characteristic residues are identified as having 1) low overall family relative entropy defined as:

Σp_i_ log_2_(p_i_/q_i_)

where for each of the 20 amino acids p_i_ is the fraction of residue type i at that alignment position, and q_i_ is the fraction of residue type i expected in a random sequence. q_i_ is usually taken from an appropriate non-redundant database and 2) high group cross-entropy computed as:

Σ(p_i_ - q_i_) log_2_(p_i_/q_i_)

where p_i_ is the fraction of residue type i at a particular position in the alignment for sequences in the predefined group while q_i_ is the fraction of residue type i at that position for sequences not in the predefined group. Often these group-specific residues are most responsible for changes in biochemical properties like substrate selectivity. We have observed in several analyses of protein families that group-specific residues often cluster around highly conserved active site residues, but that some can be quite distant which strongly suggests some stability-function tradeoff relationship. By probing the order that these residues may have appeared in their respective lineage yields insights into the how enzymes may evolve novel functions. Other programs that identify specificity determining positions though the algorithmic details are different include SDPFox [59], SPEER [60], and multi-Harmony [61]. The programs also differ in the way they treat columns in the MSA that contain gaps. Another program often highlighted in these discussions is Evolutionary Trace [62] though this program does not appear to distinguish between identifying strictly conserved and specificity-determining residues. It should be emphasized that these programs should be viewed as hypothesis generation devices since what properties constitute a group can change depending on the question you ask (see section on Sesquiterpene Synthases).

## Hybrid Quantum Chemical/Molecular Mechanical Methods

**Figure 1:**
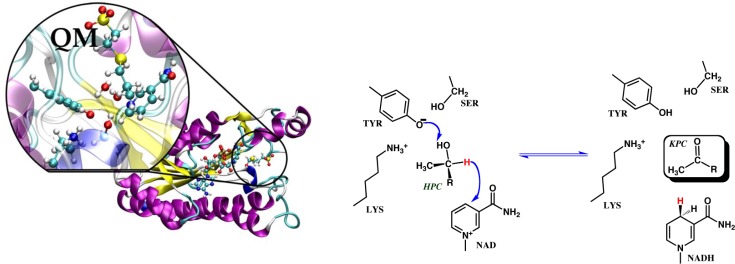
**(left)** Depiction of the R-hydroxypropylthioethanesulfonate dehydrogenase (R-HPCDH) structure with those atoms most likely to undergo significant electronic reorganization or significantly contribute to this reorganization represented by a QM method (Ser142, Tyr155, Lys140, the R-HPC substrate and the nicotinamide moiety of nicotinamide adenine dinucleotide (NAD)) while the surroundings are represented by a MM force field. Solvent molecules not shown for clarity. **(right)** Chemical mechanism for the oxidation of R-HPC by R-HPCDH.

Hybrid QC/MM potentials were first described by Warshel and Levitt [63] in a simulation of the lysozyme reaction. They were developed and continue to be extensively used to investigate the atomic and sub-atomic details of enzymatic reactions due to the high computational cost of QC methods, which are necessary for accurately modeling the electronic reorganization that occurs upon chemical bonds being broken and created, but with a goal of accurately treating the heterogeneous environment of the active site. The total potential energy in these simulations is the sum of three terms; one for the atoms in the QC region, one for those in the surrounding MM region and a term that describes the interactions between the two (see Figure 1). The methods have been the subject of numerous excellent reviews [64–67]. Despite their more widespread use, numerous challenges remain, including finding an appropriate model chemistry that can accurately represent the large (by QC standards) enzyme “active site” and a definition of the reaction coordinate. Furthermore, if free energy profiles/surfaces obtained by umbrella-sampled molecular dynamics simulations along a reaction coordinate(s) are desired, then some compromises in the QC potential must be made. Usually this requires the employment of semi empirical molecular orbital (SMO) methods [68] that are computationally efficient due to approximations of many two-electron integrals and the representation of valence electrons only. Other approximate methods based on Density Functional Theory (DFT) are also often employed [69]. All of these methods can suffer from the lack of quantitative accuracy that may be needed in order to distinguish one mechanism from another [70]. A well-established method for correcting the free energy profiles/surfaces derived from hybrid SMO/MM simulations that employs higher level QC results as a reference and spline functions that interpolate between the low and high level methods [71, 72] and which has been demonstrated to improve the free energy profiles/surfaces and result in calculated rate constants in good agreement with experiment.

Two of the major challenges in determining an enzymatic mechanism through QC/MM simulations, besides those already mentioned, is 1) representing the correct protonation states of active site residues in the Michaelis-Menten complex and 2) simulating low-barrier proton transfer reactions that may have a nuclear dynamical or tunneling component [73] and that may occur either step-wise or concerted with nucleophilic attacks. To overcome this challenge, neutron diffraction experiments of protein crystals can aid in the assignment of protonation states and the dissection of enzyme mechanistic details surrounding the location of hydrogen atoms [74]. Implicit solvent models such as MM-Poisson-Boltzmann, MM-Generalized Born [75] as well as knowledge based potentials [76] can be used for assigning protonation states. If the goal in enzyme engineering is to enhance the capabilities of natural enzymes, then dissecting the atomic details of enzymatic mechanisms will be critical to the effort.

For QC/MM simulations, our lab (TW) uses pDynamo [77] (www.pdynamo.org). This program and its Fortran predecessor was developed by Martin J. Field (Institut de Biologie Structurale) and has been utilized by our group for over 10 years to study detailed enzyme reactions. The program can directly read molecular systems constructed within CHARMM [78], AMBER [79] and GROMACS [80]. The program contains all of the standard and new semiempirical molecular orbital (SMO) methods. In addition, it contains an intuitive interface to the QC program ORCA [81] enabling higher level QC methods to be utilized for high resolution refinement or corrections to surfaces calculated with less accurate methods. Nowadays, most modern QC and MM software packages contain the capabilities to perform QC/MM simulations in some form.

## Sesquiterpene synthases

**Figure 2:**
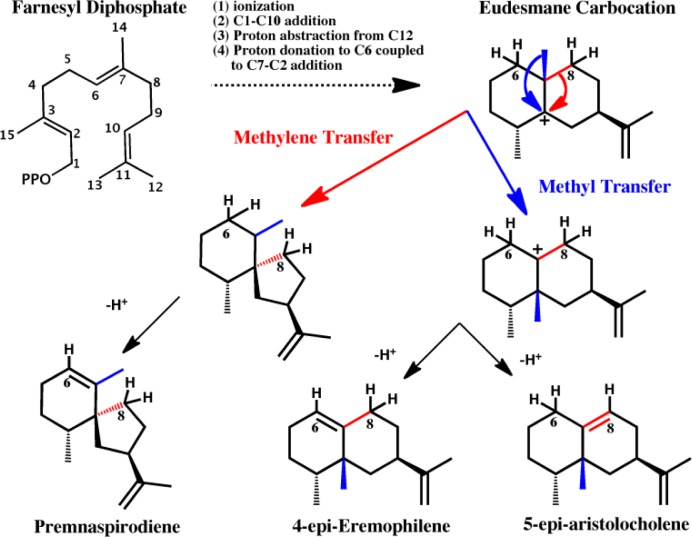
Chemical reactions progressing from the shared eudesmane carbocation intermediate to premnaspirodiene (methylene transfer followed by proton abstraction from C6), 4-epi-eremophilene and 5-epi-aristolocholene (methyl transfer followed by proton abstraction from C6 or C8 respectively).

Plant sesquiterpene synthases, a subset of the terpene synthase superfamily, are a mechanistically diverse family of enzymes capable of synthesizing hundreds of complex compounds with high regio- and stereospecificity and are of biological importance due to their role in plant defense mechanisms. Several excellent reviews on the larger terpene synthase family are available covering the essential enzymatic transformations, structural biology and phylogenetics [82–85]. Sesquiterpene synthases bind farnesyl diphosphate and three divalent Mg^2+^ ions. Sesquiterpene biosynthesis is initiated by ionization of the C1-OPP bond generating a reactive carbocation (see Figure 2 for a depiction of the unfolded form) and the first major important branching of a mechanistic network. At this point, the diphosphate moiety can then bind to C3, isomerize about the C2-C3 bond, and then ionize the C3-OPP bond to form the reactive nerolidyl carbocation. Carbocations can be quenched by proton transfer from the intermediate to the enzyme or by addition of water molecules at any point along the mechanistic decision network. Otherwise, the synthesis can proceed through an intermolecular electrophilic attack on one of the two double bonds of the substrate to form a cyclic species. Possible subsequent reactions include hydride shifts, proton transfers, methyl or methylene shifts, and further intermolecular electrophilic additions. Sesquiterpene synthases span a large range of specificity. Increasingly, similar catalytic versatility is being discovered in several protein families and may be an inherent property of enzymes [86, 87]. Such secondary catalytic activities can become the primary activity through gene duplication and subsequent divergence as well as the starting points for directed evolution in protein engineering applications [15]. Understanding these secondary activities may help lower attrition rates in drug discovery programs and identify drug interaction surfaces less susceptible to escape mutations [88].

**Figure 3:**
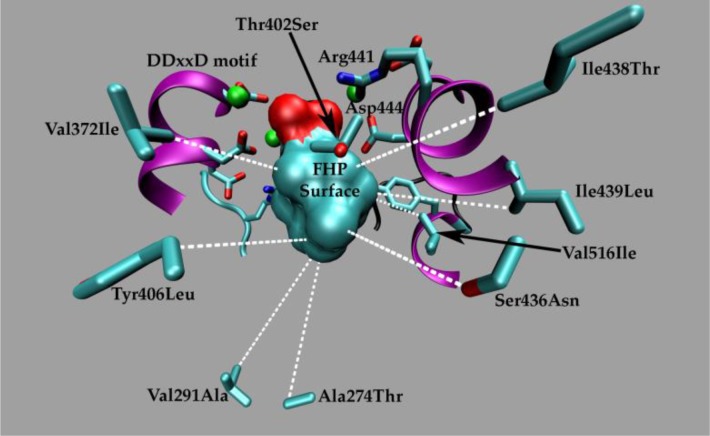
The nine residues and their substitutions located outside the active site designated here by the farnesylhydroxyphosphonate (FHP) surface and other highly conserved residues that functionally convert a 5-epi-aristolocholene synthase to a premnaspirodiene synthase.

In a recent study of sesquiterpene synthases (that encompasses all the hallmarks of the classical molecular evolution studies previously noted) in which a 5-epi-aristolocholene synthase (5EAS) was transformed to a premnaspirodiene synthase (PSDS) through mutational swaps of nine residues (none of which make specific contacts with the substrate) as well as experimental classification of all 512 proteins with different combinations of these nine residues, a functional landscape underlying the evolution of sesquiterpene chemical diversity was revealed (see Figures 2 and 3) [89]. The catalytic cycle of both synthases passes through several common intermediate states and only diverges in the last few chemical steps with some mutants along this putative evolutionary swath producing 4-epi-eremophilene, a product with hybrid activity; possibly ancestral to both enzymes. Also of significance, the mutants exhibited functional epistasis by the fact that 1) no single amino acid correlated with the product distribution and 2) the effect of a mutation depended on the state of the other eight residues. While these studies on sesquiterpene synthases are highly innovative and relevant to the task of seeking fundamental enzyme design principles, a decisive physicochemical explanation for the evolution of novel sesquiterpene synthase function is lacking. Therefore, our lab began investigating these details by first performing an extensive molecular phylogenetic analysis and then leveraging this information to support mechanistic conclusions obtained from atomic MD simulations [2]. Uncovering the biophysical principles governing a functional landscape can assist in narrowing the many possible evolutionary pathways to novel functions [9]; information that deepens our knowledge of structure-function relationships.

**Figure 4:**
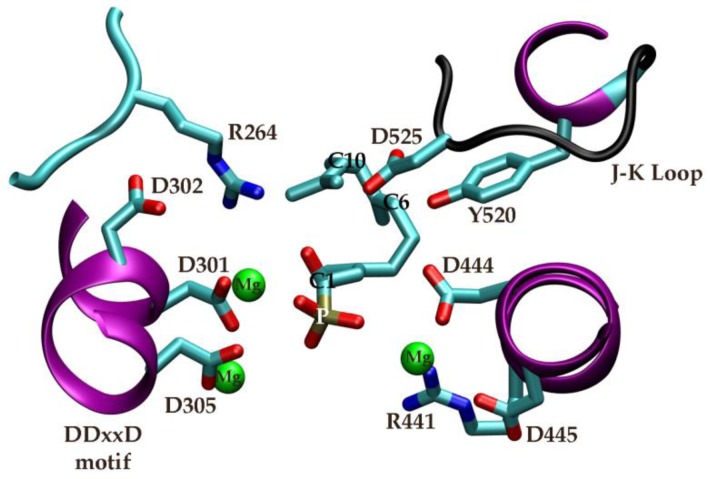
Highly conserved (<95%) residues highlighted on the 5-epi-aristolocholene synthase active site structure (PDB entry: 5eat). Also shown are the co-crystallized Mg^2+^ ions (green) and farnesylhydroxyphosphanate substrate mimic (middle). Helices are in purple. Reprinted from Reference 2 with permission.

Our recently published molecular phylogenetic analysis of the plant sesquiterpene synthase family has been, and can in the future, be utilized to support conclusions from the simulations and to leverage experimental results on sesquiterpene synthases from other branches of the phylogenetic tree [2]. The number and complexity of sesquiterpene products had in the past inhibited the use of phylogenetic analysis for constructing sequence-function relationships because of uncertainties in the statistical relevance of the resulting inferences. However, through a carefully-crafted multiple sequence alignment of ∼200 plant sesquiterpene synthases using the procedures described in the Molecular Phylogenetics section, we observed that all sequences that cluster together on the phylogenetic tree into well-defined groups share at least the first reaction in the catalytic mechanism subsequent to the initial ionization step and many share steps beyond this, down to proton transfers between the enzyme and substrate. The multiple sequence alignment showed 15 highly conserved residues (95% and above) in the C-terminal catalytic domain, five of which are outside the active site. Most significant was the previously unreported high conservation of a Tyr520-Asp444-Asp525 triad (numbering according to the Nicotiana tobacum sequence for which there is a structure, see Figure 4). Given the high conservation of the Asp444-Tyr-520-Asp-525 triad, its position relative to a folded substrate analogue [90], the demonstration of its importance in generating (+)-germacrene A either as an intermediate or product [91], and our own atomistic MD simulations of the eudesmane carbocation in 5EAS, and finally the absence of likely proton donors/acceptors in other parts of many plant sTS active sites, we proposed that this triad is an important functional element responsible for many proton transfers to and from the substrate and intermediates along the plant sesquiterpene synthase catalytic cycle. Though this triad is obviously not the key to understanding all of plant sTS enzymatic chemistry, we nevertheless proposed that the triad can be tuned in a variety of ways to generate a diversity of products. These include 1) substituting residues on the opposite side of the active site forcing the farnesyl diphosphate to fold in different ways so that through both mechanisms the triad will donate/abstract protons to and from different carbons and 2) substituting residues surrounding the triad to shift their position relative to the substrate and/or intermediates. Finally, these results highlight what insights can be gained and a wealth of hypotheses that can arise from a phylogenetic analysis coupled to molecular modeling of the substrate/intermediate-enzyme complex. Developing these hypotheses was critical to our efforts to dissect the 5EAS mechanism further by hybrid QC/MM methods.

The first structural models of a plant sesquiterpene synthase [90] based on x-ray crystallography presented enough information to propose a full mechanism for the many chemical steps in transforming farnesyl pyrophosphate to its product, 5-epi-aristolocholene (5EA). Part of this mechanism has gained experimental support [91], though the latter steps that form the basis of functional divergence remain to be fully explained. Furthermore, much is still unknown about the atomic details of many sesquiterpene synthase mechanisms, even though the intrinsic reactivity of the substrate has in several cases been elucidated through quantum chemical calculations [92, 93]. A QC/MM simulation study on a fungal aristolocholene synthase discovered an energetically feasible intramolecular proton transfer reaction leading to formation of the eudesmane carbocation as well as determining the functional roles of active site residues [94]. Another QC/MM study on a closely related bornyl diphosphate synthase revealed how the electrostatic environment of the enzyme's active site steered the substrate to its’ final product [95]. Due to the transient nature of so many intermediate states along terpene synthase catalytic cycles, it is extremely difficult to experimentally determine reaction rates or thermodynamic data for the individual reactions. In addition, simulation studies on terpene synthases are quite challenging due to the fact that the enzymes’ contribution to catalysis may be relatively subtle with residues appearing to act in concert and ones outside the active site affecting product distributions [89]. Past QC calculations on the methyl and methylene transfer reactions emanating from the eudesmane carbocation in 5EAS [96] have primarily served to amplify the mystery behind the physicochemical properties that control sesquiterpene synthase product distributions. These reactions could be under thermodynamic or kinetic control. The enzyme may preferentially stabilize the non-classical carbocation (carbonium ions) structures that occur along both of these reaction paths. Yet, our latest simulation results (manuscript in preparation) employing QC/MM methods and representation of the fully solvated 5EAS-eudesmane carbocation intermediate reveals the basis for 5EAS specificity is the preferential stabilization of the 4-epi-eremophilenyl intermediate (see Figure 2) over the premnaspirodienyl intermediate. The calculated free energy barrier for both reactions is very low, around 5-6 kcal/mol. The free energy differences, on the other hand, are exergonic for the methyl transfer reaction and endergonic for the methylene transfer. The methyl transfer reaction from the eudesmane carbocation is favored in 5EAS in part because the active site forms a “cage” around the substrate's isoprenyl group which is not “disturbed” by the methyl transfer reaction. In contrast, the methylene transfer reaction results in a more substantial change in the shape of the intermediate and several steric clashes occur between the isoprenyl group of the substrate and surrounding residues. Furthermore, the catalytic triad of Asp444-Tyr520-Asp525 is favorably positioned to abstract the proton from C8 of the 4-epi-eremophilenyl carbocation resulting in 5EA product formation. Thus, there are multiple structural and functional requirements for specificity to arise in 5EAS. Further examination of this functional landscape [89] through hybrid QC/MM simulations could provide direction on how to engineer these enzymes to be less promiscuous and biosynthesize completely new natural products.

## Class D β-lactamases

**Figure 5:**
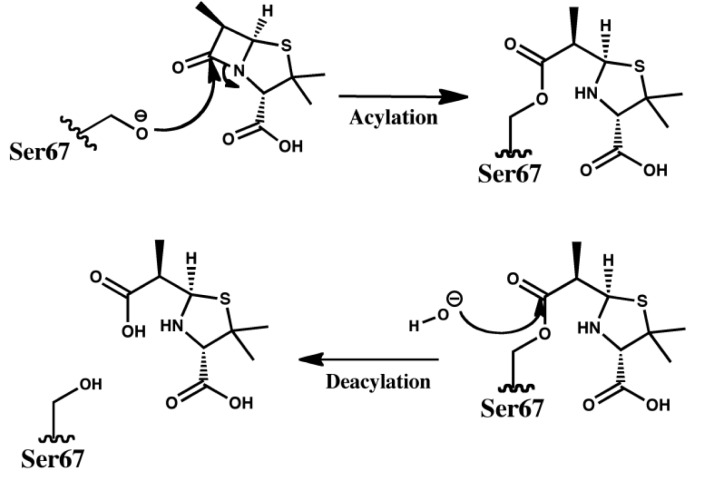
Two stage process for the hydrolysis of antibiotic molecule by β-lactamases.

Because of their safety and efficacy, β-lactams still constitute the most widely used antibiotics that inhibit bacterial cell-wall transpeptidases (PBPs – Penicilin Binding Proteins). Bacteria's most important resistance mechanism relies on the scavenging potential of β-lactamases to break the amide bond before antibiotics reach their cellular target. The reaction is a two-step process: 1) acylation of the enzyme Ser residue followed by 2) deacylation and release of the cleaved antibiotic (see Figure 5). Phylogenetic analysis divides the β-lactamases into groups A, C, D (serine hydrolases) and B (metallo- enzymes). The remarkable variety of β-lactamases, the rapid rate of their evolution and acquisition of resistance towards newly developed drugs make it of prime importance for us to understand fully their sequence–structure–function relationships and evolutionary mechanisms towards antibiotic resistance in order to break the current cycle of drug development followed by resistance. Class D, often referred to as OXA, after several initially classified members demonstrated unusually high hydrolysis efficacy against oxacillin, is the most diverse group of β-lactamases, most recently identified, and also the least studied [97, 98]. They possess a remarkably diverse range of hydrolytic profiles encompassing penicillins, cephems (including third generation drugs: cefotaxime and ceftazidime) as well as carbapenems [98]. Interestingly, however, to date no class D enzyme has shown ability to hydrolyze both extended spectrum cephalosporins and carbapenems [98, 99].

**Figure 6:**
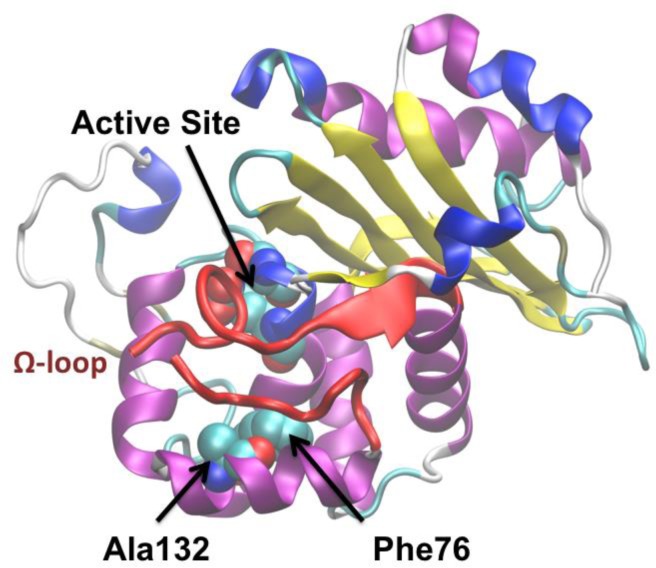
“OXA-2” group-specific residues (Phe76 and Ala132) within the OXA-2 structure (PDB entry 1K38)

Class D is one of two known enzymes that employ a post-translational carboxylation of a lysine residue as part of their catalytic machinery. This carboxylated lysine (Lys70) is crucial for activating a water molecule for the deacylation reaction and likely acts as a general base to abstract a proton from the absolutely conserved Ser67 that initiates nucleophilic attack on the β-lactam ring. Our recently published phylogenetic analysis of class D enabled the division/classifying of the sequences into distinct groups [3]. Pairwise sequence identities within these groups were very small (as low as 22% in the largest group), yet there were distinct signatures or group-specific residues that allowed us to uniquely demarcate the groups. These group-specific residues are mainly located adjacent to the active site with their van der Waals surfaces in contact with the binding pocket. Thus they are likely to modulate the properties of the conserved active site catalytic residues [21, 100] and may correlate with the enzyme's spectrum of activity (see Figure 6). In addition, their location outside the active site may have only a minimal effect on protein stability [16]. The largest sub-group of class D sequences (OXA-1 subgroup) made up of α-, β-, and γ-proteobacteria also contains a group-specific Cys pair. In the OXA-1 crystal structure (PDB entry 3ISG [101]) this Cys pair is positioned to form a disulfide bond and our analysis of the deposited electron density map reveals that both the reduced and oxidized forms are present in apo OXA-1 (PDB entry 1M6K [102]). The reducing cytoplasmic environment of the *E. coli* expression system is responsible for partial or complete reduction of the disulfide bond and its apparent absence in the OXA-1 3ISG crystal structure. Patterns of evolutionary constraint seen in our analyses support the prediction that formation of this disulfide bond in an oxidizing environment may be important for survival by maintaining the hydrophobic core of the enzyme. Additional support to this hypothesis is provided by the fact that *P. aeruginosa* group (OXA- 10 group) also contains a group-specific disulfide-bonded Cys pair – located on the opposite end of the same segment of β-sheet (PDB entry 1K57 [103]). Alternatively, the increased stabilization of this region may have an impact on the dynamic behavior of the active site. Long timescale (several microseconds) MD simulations using classical MM force fields showed that when the Cys pair is in a reduced state, the active site becomes disorganized which if accurate would also lead to decarboxylation of the Lys sidechain and destruction of the enzyme's function (manuscript in preparation) while in the oxidized state the active site remained stable.

## Summary and Outlook

The mechanistic details of enzymatic reactions obtained from hybrid QC/MM simulations combined with identification of amino acid residues critical for the maintenance and diversity of function within a enzyme (super)family is a powerful approach for elucidating enzyme design principles. This information can then be leveraged to design enzymes for environmental remediation, energy production, the prediction of antibiotic resistance and the production of therapeutic compounds. The use of QC/MM methods to investigate enzymatic reactions still requires some expertise. The resulting free energy profiles or surfaces can be very sensitive to the initial coordinates, protonation states of surrounding residues, the QC method, and the length of umbrella sampling simulations [104]. Nevertheless, there have been significant improvements in software employing QC/MM methods over the last ten years since the authors began using them. We can expect to see further improvement in semiempirical molecular orbital parameterizations and methods which will enable faster exploration of possible reactive configurations as well as the generation of initial reaction paths that can be refined with higher level QC methods. But possibly more importantly is that in the future we will increasingly see the atomic and subatomic level details of enzyme reaction mechanisms interpreted within the context of its evolutionary history using molecular phylogenetics. One of the main limitations in this endeavor today is the fact that so many sequences are uncharacterized or even mischaracterized [105]. Assignment of function by homology may only be successful at relatively high sequence identities. Beyond this, the enzyme may carry out essentially the same catalytic chemistry but on very different substrates. Until this problem is remedied, the value of the sequence data will not reach its full potential benefit for use in enzyme design. For example, if a much larger percentage of plant sesquiterpene synthase sequences had experimentally determined functions, then analyses could be performed on the MSA to determine what residues are most responsible for traversing the nerolidyl pathway versus the trans pathway. Nevertheless, these are exciting times for computational biochemists who have an amazing opportunity to utilize both atomic-scale MD simulations, using both MM and QC/MM potential energy functions, and molecular phylogenetics in their research that will provide results and subsequent stories (publications), one could argue, that can stand aside some of the past classical studies of adaptation and begin to provide robust answers to the challenging questions posed in this review.

## References

[1] Harms MJ, Thornton JW (2010) Analyzing protein structure and function using ancestral gene reconstruction. Curr Opin Struct Biol. 10.1016/j.sbi.2010.03.005 PMC2916957 20413295

[2] Wymore, T, Chen, BY, Nicholas, HB, Ropelewski, AJ, Brooks, CL (2011) A Mechanism for Evolving Novel Plant Sesquiterpene Synthase Function. Molecular Informatics 30: 896–906 10.1002/minf.201100087 27468109

[3] Szarecka, A, Lesnock, KR, Ramirez-Mondragon, CA, Nicholas, HB, Wymore, T (2011) The Class D β-lactamase family: residues governing the maintenance and diversity of function. Protein Engineering Design and Selection 24: 801–809 10.1093/protein/gzr041 PMC3170078 21859796

[4] Glasner, ME, Gerlt, JA, Babbitt, PC (2006) Evolution of enzyme superfamilies. Current opinion in chemical biology 10: 492–497 16935022 10.1016/j.cbpa.2006.08.012

[5] Hartley, CJ, Newcomb, RD, Russell, RJ, Yong, CG, Stevens, JR, et al. (2006) Amplification of DNA from preserved specimens shows blowflies were preadapted for the rapid evolution of insecticide resistance. Proc Natl Acad Sci USA 103: 8757–8762 16723400 10.1073/pnas.0509590103 PMC1482651

[6] Shi, Y, Yokoyama, S (2003) Molecular analysis of the evolutionary significance of ultraviolet vision in vertebrates. Proc Natl Acad Sci USA 100: 8308–8313 12824471 10.1073/pnas.1532535100 PMC166225

[7] Bershtein, S, Goldin, K, Tawfik, DS (2008) Intense neutral drifts yield robust and evolvable consensus proteins. J Mol Biol 379: 1029–1044 18495157 10.1016/j.jmb.2008.04.024

[8] Wang, X, Minasov, G, Shoichet, BK (2002) Evolution of an antibiotic resistance enzyme constrained by stability and activity trade-offs. J Mol Biol 320: 85–95 12079336 10.1016/S0022-2836(02)00400-X

[9] Weinreich, DM, Delaney, NF, Depristo, MA, Hartl, DL (2006) Darwinian evolution can follow only very few mutational paths to fitter proteins. Science 312: 111–114 16601193 10.1126/science.1123539

[10] Lunzer, M, Miller, SP, Felsheim, R, Dean, AM (2005) The biochemical architecture of an ancient adaptive landscape. Science 310: 499–501 16239478 10.1126/science.1115649

[11] Miller, SP, Lunzer, M, Dean, AM (2006) Direct demonstration of an adaptive constraint. Science 314: 458–461 17053145 10.1126/science.1133479

[12] Bridgham, JT, Carroll, SM, Thornton, JW (2006) Evolution of hormone-receptor complexity by molecular exploitation. Science 312: 97–101 37000978 10.1681/01.asn.0000926836.46869.e5

[13] Ortlund, EA, Bridgham, JT, Redinbo, MR, Thornton, JW (2007) Crystal structure of an ancient protein: evolution by conformational epistasis. Science 317: 1544–1548 17702911 10.1126/science.1142819 PMC2519897

[14] Bridgham, JT, Ortlund, EA, Thornton, JW (2009) An epistatic ratchet constrains the direction of glucocorticoid receptor evolution. Nature 461: 515–519 19779450 10.1038/nature08249 PMC6141187

[15] Romero, PA, Arnold, FH (2009) Exploring protein fitness landscapes by directed evolution. Nat Rev Mol Cell Biol 10: 866–876 19935669 10.1038/nrm2805 PMC2997618

[16] Depristo, MA, Weinreich, DM, Hartl, DL (2005) Missense meanderings in sequence space: a biophysical view of protein evolution. Nat Rev Genet 6: 678–687 16074985 10.1038/nrg1672

[17] Tokuriki, N, Tawfik, DS (2009) Stability effects of mutations and protein evolvability. Current Opinion in Structural Biology 19: 596–604 19765975 10.1016/j.sbi.2009.08.003

[18] Tokuriki, N, Stricher, F, Serrano, L, Tawfik, DS (2008) How protein stability and new functions trade off. PLoS Computational Biology 4: e1000002 18463696 10.1371/journal.pcbi.1000002 PMC2265470

[19] Conant, GC, Wolfe, KH (2008) Turning a hobby into a job: how duplicated genes find new functions. Nat Rev Genet 9: 938–950 19015656 10.1038/nrg2482

[20] Poelwijk, FJ, Kiviet, DJ, Weinreich, DM, Tans, SJ (2007) Empirical fitness landscapes reveal accessible evolutionary paths. Nature 445: 383–386 17251971 10.1038/nature05451

[21] Phillips, PC (2008) Epistasis--the essential role of gene interactions in the structure and evolution of genetic systems. Nat Rev Genet 9: 855–867 18852697 10.1038/nrg2452 PMC2689140

[22] ZUCKERKANDL E (1976) Evolutionary processes and evolutionary noise at the molecular level. I. Functional density in proteins. J Mol Evol 7: 167–183 933174 10.1007/BF01731487

[23] Smith, JM (1970) Natural selection and the concept of a protein space. Nature 225: 563–564 5411867 10.1038/225563a0

[24] Dean, AM, Thornton, JW (2007) Mechanistic approaches to the study of evolution: the functional synthesis. Nat Rev Genet 8: 675–688 17703238 10.1038/nrg2160 PMC2488205

[25] Stern, DL, Orgogozo, V (2009) Is genetic evolution predictable?. Science 323: 746–751 19197055 10.1126/science.1158997 PMC3184636

[26] Laskowski R, Gerick F, Thornton J (2009) The structural basis of allosteric regulation in proteins. FEBS Letters 10.1016/j.febslet.2009.03.019 19303011

[27] Yoshikuni, Y, Dietrich, JA, Nowroozi, FF, Babbitt, PC, Keasling, JD (2008) Redesigning enzymes based on adaptive evolution for optimal function in synthetic metabolic pathways. Chemistry & Biology 15: 607–618 18559271 10.1016/j.chembiol.2008.05.006 PMC4030648

[28] Warshel, A (2003) Computer simulations of enzyme catalysis: methods, progress, and insights. Annu Rev Biophys Biomol Struct 32: 425–443 12574064 10.1146/annurev.biophys.32.110601.141807

[29] Smith, AJT, Müller, R, Toscano, MD, Kast, P, Hellinga, HW, et al. (2008) Structural reorganization and preorganization in enzyme active sites: comparisons of experimental and theoretically ideal active site geometries in the multistep serine esterase reaction cycle. Journal of the American Chemical Society 130: 15361–15373 18939839 10.1021/ja803213p PMC2728765

[30] Boehr, DD, Nussinov, R, Wright, PE (2009) The role of dynamic conformational ensembles in biomolecular recognition. Nat Chem Biol 5: 789–796 19841628 10.1038/nchembio.232 PMC2916928

[31] Goodey, NM, Benkovic, SJ (2008) Allosteric regulation and catalysis emerge via a common route. Nat Chem Biol 4: 474–482 18641628 10.1038/nchembio.98

[32] Cui, Q, Karplus, M (2008) Allostery and cooperativity revisited. Protein Sci 17: 1295–1307 18560010 10.1110/ps.03259908 PMC2492820

[33] Hassan, A, Koh, J (2008) Selective Chemical Rescue of a Thyroid-Hormone-Receptor Mutant, TRbeta(H435Y), Identified in Pituitary Carcinoma and Resistance to Thyroid Hormone. Angew Chem Int Ed Engl 47: 7280–7283 18683837 10.1002/anie.200801742 PMC11556420

[34] Chen, C-H, Budas, GR, Churchill, EN, Disatnik, M-H, Hurley, TD, et al. (2008) Activation of aldehyde dehydrogenase-2 reduces ischemic damage to the heart. Science 321: 1493–1495 18787169 10.1126/science.1158554 PMC2741612

[35] Wagner, A (2008) Robustness and evolvability: a paradox resolved. Proc Biol Sci 275: 91–100 17971325 10.1098/rspb.2007.1137 PMC2562401

[36] Whelan, S, Liò, P, Goldman, N (2001) Molecular phylogenetics: state-of-the-art methods for looking into the past. Trends Genet 17: 262–272 11335036 10.1016/s0168-9525(01)02272-7

[37] Altschul, SF, Gish, W, Miller, W, Myers, EW, Lipman, DJ (1990) Basic local alignment search tool. Journal of Molecular Biology 215: 403–410 2231712 10.1016/S0022-2836(05)80360-2

[38] Altschul, SF, Madden, TL, Schaffer, AA, Zhang, J, Zhang, Z, et al. (1997) Gapped BLAST and PSI-BLAST: a new generation of protein database search programs. Nucleic acids research 25: 3389–3402 9254694 10.1093/nar/25.17.3389 PMC146917

[39] Wu, CH, Huang, H, Nikolskaya, A, Hu, Z, Barker, WC (2004) The iProClass integrated database for protein functional analysis. Comput Biol Chem 28: 87–96 15022647 10.1016/j.compbiolchem.2003.10.003

[40] Huang, Y, Niu, B, Gao, Y, Fu, L, Li, W (2010) CD-HIT Suite: a web server for clustering and comparing biological sequences. Bioinformatics 26: 680–682 20053844 10.1093/bioinformatics/btq003 PMC2828112

[41] Notredame, C, Higgins, DG, Heringa, J (2000) T-Coffee: A novel method for fast and accurate multiple sequence alignment. Journal of Molecular Biology 302: 205–217 10964570 10.1006/jmbi.2000.4042

[42] Edgar, RC (2004) MUSCLE: multiple sequence alignment with high accuracy and high throughput. Nucleic Acids Res 32: 1792–1797 15034147 10.1093/nar/gkh340 PMC390337

[43] Do, CB, Mahabhashyam, MS, Brudno, M, Batzoglou, S (2005) ProbCons: Probabilistic consistency-based multiple sequence alignment. Genome Research 15: 330–340 15687296 10.1101/gr.2821705 PMC546535

[44] Larkin, MA, Blackshields, G, Brown, NP, Chenna, R, McGettigan, PA, et al. (2007) Clustal W and Clustal X version 2.0. Bioinformatics 23: 2947–2948 17846036 10.1093/bioinformatics/btm404

[45] Edgar, RC, Batzoglou, S (2006) Multiple sequence alignment. Curr Opin Struct Biol 16: 368–373 16679011 10.1016/j.sbi.2006.04.004

[46] Procter, JB, Thompson, J, Letunic, I, Creevey, C, Jossinet, F, et al. (2010) Visualization of multiple alignments, phylogenies and gene family evolution. Nature methods 7: S16–25 20195253 10.1038/nmeth.1434

[47] Waterhouse, AM, Procter, JB, Martin, DMA, Clamp, M, Barton, GJ (2009) Jalview Version 2--a multiple sequence alignment editor and analysis workbench. Bioinformatics 25: 1189–1191 19151095 10.1093/bioinformatics/btp033 PMC2672624

[48] Bailey TL (1994) Fitting a mixture model by expectation maximization to discover motifs in biopolymers. Proceedings of 2nd International Conference on ISMB: 1–7 7584402

[49] Roberts, E, Eargle, J, Wright, D, Luthey-Schulten, Z (2006) MultiSeq: unifying sequence and structure data for evolutionary analysis. BMC Bioinformatics 7: 382 16914055 10.1186/1471-2105-7-382 PMC1586216

[50] Humphrey, W, Dalke, A, Schulten, K (1996) VMD: visual molecular dynamics. Journal of molecular graphics 14: 33–38, 27-38 8744570 10.1016/0263-7855(96)00018-5

[51] Hasegawa, H, Holm, L (2009) Advances and pitfalls of protein structural alignment. Current Opinion in Structural Biology 19: 341–348 19481444 10.1016/j.sbi.2009.04.003

[52] Sjölander, K (2004) Phylogenomic inference of protein molecular function: advances and challenges. Bioinformatics 20: 170–179 14734307 10.1093/bioinformatics/bth021

[53] Sjölander, K (2010) Getting started in structural phylogenomics. PLoS Computational Biology 6: e1000621 20126522 10.1371/journal.pcbi.1000621 PMC2813252

[54] Capella-Gutierrez, S, Silla-Martinez, JM, Gabaldon, T (2009) trimAl: a tool for automated alignment trimming in large-scale phylogenetic analyses. Bioinformatics 25: 1972–1973 19505945 10.1093/bioinformatics/btp348 PMC2712344

[55] Talavera, G, Castresana, J (2007) Improvement of phylogenies after removing divergent and ambiguously aligned blocks from protein sequence alignments. Systematic biology 56: 564–577 17654362 10.1080/10635150701472164

[56] Thornton, JW (2004) Resurrecting ancient genes: experimental analysis of extinct molecules. Nat Rev Genet 5: 366–375 15143319 10.1038/nrg1324

[57] Casari, G, Sander, C, Valencia, A (1995) A method to predict functional residues in proteins. Nature structural biology 2: 171–178 10.1038/nsb0295-171 7749921

[58] Maetschke, SR, Kassahn, KS, Dunn, JA, Han, S-P, Curley, EZ, et al. (2010) A visual framework for sequence analysis using n-grams and spectral rearrangement. Bioinformatics 26: 737–744 20130028 10.1093/bioinformatics/btq042

[59] Mazin, PV, Gelfand, MS, Mironov, AA, Rakhmaninova, AB, Rubinov, AR, et al. (2010) An automated stochastic approach to the identification of the protein specificity determinants and functional subfamilies. Algorithms for molecular biology: AMB 5: 29 20633297 10.1186/1748-7188-5-29 PMC2914642

[60] Chakraborty, A, Mandloi, S, Lanczycki, CJ, Panchenko, AR, Chakrabarti, S (2012) SPEER-SERVER: a web server for prediction of protein specificity determining sites. Nucleic acids research 40: W242–248 22689646 10.1093/nar/gks559 PMC3394334

[61] Brandt, BW, Feenstra, KA, Heringa, J (2010) Multi-Harmony: detecting functional specificity from sequence alignment. Nucleic acids research 38: W35–40 20525785 10.1093/nar/gkq415 PMC2896201

[62] Lichtarge, O, Bourne, HR, Cohen, FE (1996) An evolutionary trace method defines binding surfaces common to protein families. Journal of Molecular Biology 257: 342–358 8609628 10.1006/jmbi.1996.0167

[63] Warshel, A, Levitt, M (1976) Theoretical studies of enzymic reactions: dielectric, electrostatic and steric stabilization of the carbonium ion in the reaction of lysozyme. Journal of Molecular Biology 103: 227–249 985660 10.1016/0022-2836(76)90311-9

[64] Gao, J, Ma, S, Major, DT, Nam, K, Pu, J, et al. (2006) Mechanisms and free energies of enzymatic reactions. Chem Rev 106: 3188–3209 16895324 10.1021/cr050293k PMC4477011

[65] Hu, H, Yang, W (2008) Free energies of chemical reactions in solution and in enzymes with ab initio quantum mechanics/molecular mechanics methods. Annu Rev Phys Chem 59: 573–601 18393679 10.1146/annurev.physchem.59.032607.093618 PMC3727228

[66] Mulholland, AJ (2005) Modelling enzyme reaction mechanisms, specificity and catalysis. Drug Discov Today 10: 1393–1402 16253878 10.1016/S1359-6446(05)03611-1

[67] Senn, HM, Thiel, W (2009) QM/MM methods for biomolecular systems. Angew Chem Int Ed Engl 48: 1198–1229 19173328 10.1002/anie.200802019

[68] Stewart, JJP (2007) Optimization of parameters for semiempirical methods V: modification of NDDO approximations and application to 70 elements. Journal of molecular modeling 13: 1173–1213 17828561 10.1007/s00894-007-0233-4 PMC2039871

[69] Elstner, M (2006) The SCC-DFTB method and its application to biological systems. Theor Chem Acc 116: 316–325

[70] Sattelmeyer, KW, Tirado-Rives, J, Jorgensen, WL (2006) Comparison of SCC-DFTB and NDDO-based semiempirical molecular orbital methods for organic molecules. The journal of physical chemistry A, Molecules, spectroscopy, kinetics, environment & general theory 110: 13551–13559 10.1021/jp064544k 17165882

[71] Chuang, Y, Corchado, J, Truhlar, D (1999) Mapped interpolation scheme for single-point energy corrections in reaction rate calculations and a critical evaluation of dual-level reaction path dynamics methods. J Phys Chem A 103: 1140–1149

[72] Ruiz-Pernia, J, Silla, E, Tunon, I, Marti, S, Moliner, V (2004) Hybrid QM/MM potentials of mean force with interpolated corrections. J Phys Chem B 108: 8427–8433

[73] Truhlar, DG, Gao, J, Alhambra, C, Garcia-Viloca, M, Corchado, J, et al. (2002) The incorporation of quantum effects in enzyme kinetics modeling. Acc Chem Res 35: 341–349 12069618 10.1021/ar0100226

[74] Glusker, JP, Carrell, HL, Kovalevsky, AY, Hanson, L, Fisher, SZ, et al. (2010) Using neutron protein crystallography to understand enzyme mechanisms. Acta Crystallographica Section D-Biological Crystallography 66: 1257–1261 10.1107/S0907444910027915 PMC2967424 21041947

[75] Chen, J, Brooks, CL 3rd, Khandogin, J (2008) Recent advances in implicit solvent-based methods for biomolecular simulations. Current Opinion in Structural Biology 18: 140–148 18304802 10.1016/j.sbi.2008.01.003 PMC2386893

[76] Olsson, MHM, Sondergaard, CR, Rostkowski, M, Jensen, JH (2011) PROPKA3: Consistent Treatment of Internal and Surface Residues in Empirical pK(a) Predictions. Journal of Chemical Theory and Computation 7: 525–537 10.1021/ct100578z 26596171

[77] Field, M (2008) The pDynamo Program for Molecular Simulations using Hybrid Quantum Chemical and Molecular Mechanical Potentials. Journal of Chemical Theory and Computation 4: 1151–1161 10.1021/ct800092p 26636368

[78] Brooks, BR, Brooks, CL, Mackerell, AD, Nilsson, L, Petrella, RJ, et al. (2009) CHARMM: the biomolecular simulation program. Journal of computational chemistry 30: 1545–1614 19444816 10.1002/jcc.21287 PMC2810661

[79] Case, DA, Cheatham, TE, Darden, T, Gohlke, H, Luo, R, et al. (2005) The Amber biomolecular simulation programs. Journal of computational chemistry 26: 1668–1688 16200636 10.1002/jcc.20290 PMC1989667

[80] Hess, B, Kutzner, C, van der Spoel, D, Lindahl, E (2008) GROMACS 4: Algorithms for highly efficient, load-balanced, and scalable molecular simulation. Journal of Chemical Theory and Computation 4: 435–447 10.1021/ct700301q 26620784

[81] Neese, F (2012) The ORCA program system. Wiley Interdisciplinary Reviews-Computational Molecular Science 2: 73–78

[82] Christianson, DW (2008) Unearthing the roots of the terpenome. Current Opinion in Chemical Biology 12: 141–150 18249199 10.1016/j.cbpa.2007.12.008 PMC2430190

[83] Christianson, DW (2006) Structural biology and chemistry of the terpenoid cyclases. Chem Rev 106: 3412–3442 16895335 10.1021/cr050286w

[84] CANE D (1990) ENZYMATIC FORMATION OF SESQUITERPENES. Chem Rev 90: 1089–1103

[85] Degenhardt, J, Köllner, TG, Gershenzon, J (2009) Monoterpene and sesquiterpene synthases and the origin of terpene skeletal diversity in plants. Phytochemistry 70: 1621–1637 19793600 10.1016/j.phytochem.2009.07.030

[86] Babtie, A, Tokuriki, N, Hollfelder, F (2010) What makes an enzyme promiscuous?. Current opinion in chemical biology 14: 200–207 20080434 10.1016/j.cbpa.2009.11.028

[87] Khersonsky, O, Tawfik, DS (2010) Enzyme promiscuity: a mechanistic and evolutionary perspective. Annu Rev Biochem 79: 471–505 20235827 10.1146/annurev-biochem-030409-143718

[88] Nobeli, I, Favia, AD, Thornton, JM (2009) Protein promiscuity and its implications for biotechnology. Nat Biotechnol 27: 157–167 19204698 10.1038/nbt1519

[89] O'maille, PE, Malone, A, Dellas, N, Andes Hess, B, Smentek, L, et al. (2008) Quantitative exploration of the catalytic landscape separating divergent plant sesquiterpene synthases. Nat Chem Biol 4: 617–623 18776889 10.1038/nchembio.113 PMC2664519

[90] Starks, CM, Back, K, Chappell, J, Noel, JP (1997) Structural basis for cyclic terpene biosynthesis by tobacco 5-epi-aristolochene synthase. Science 277: 1815–1820 9295271 10.1126/science.277.5333.1815

[91] Rising, K, Starks, C, Noel, J, Chappell, J (2000) Demonstration of germacrene A as an intermediate in 5-epi-aristolochene synthase catalysis. Journal of the American Chemical Society 122: 1861–1866

[92] Tantillo, DJ (2010) The carbocation continuum in terpene biosynthesis--where are the secondary cations?. Chem Soc Rev 39: 2847–2854 20442917 10.1039/b917107j

[93] Tantillo, DJ (2011) Biosynthesis via carbocations: theoretical studies on terpene formation. Natural product reports 28: 1035–1053 21541432 10.1039/c1np00006c

[94] Allemann, RK, Young, NJ, Ma, S, Truhlar, DG, Gao, J (2007) Synthetic efficiency in enzyme mechanisms involving carbocations: aristolochene synthase. Journal of the American Chemical Society 129: 13008–13013 17918834 10.1021/ja0722067 PMC2528250

[95] Weitman, M, Major, DT (2010) Challenges posed to bornyl diphosphate synthase: diverging reaction mechanisms in monoterpenes. Journal of the American Chemical Society 132: 6349–6360 20394387 10.1021/ja910134x

[96] Hess, BAJr, Smentek, L, Noel, JP, O'Maille, PE (2011) Physical constraints on sesquiterpene diversity arising from cyclization of the eudesm-5-yl carbocation. Journal of the American Chemical Society 133: 12632–12641 21714557 10.1021/ja203342p

[97] Fisher, JF, Meroueh, SO, Mobashery, S (2005) Bacterial resistance to beta-lactam antibiotics: compelling opportunism, compelling opportunity. Chem Rev 105: 395–424 15700950 10.1021/cr030102i

[98] Poirel, L, Naas, T, Nordmann, P (2010) Diversity, epidemiology, and genetics of class D beta-lactamases. Antimicrobial Agents and Chemotherapy 54: 24–38 19721065 10.1128/AAC.01512-08 PMC2798486

[99] Poirel, L, Nordmann, P (2006) Carbapenem resistance in Acinetobacter baumannii: mechanisms and epidemiology. Clin Microbiol Infect 12: 826–836 16882287 10.1111/j.1469-0691.2006.01456.x

[100] Majiduddin, FK, Palzkill, T (2005) Amino acid residues that contribute to substrate specificity of class A beta-lactamase SME-1. Antimicrobial Agents and Chemotherapy 49: 3421–3427 16048956 10.1128/AAC.49.8.3421-3427.2005 PMC1196253

[101] Schneider, KD, Karpen, ME, Bonomo, RA, Leonard, DA, Powers, RA (2009) The 1.4 A crystal structure of the class D beta-lactamase OXA-1 complexed with doripenem. Biochemistry 48: 11840–11847 19919101 10.1021/bi901690r PMC2805451

[102] Sun, T, Nukaga, M, Mayama, K, Braswell, EH, Knox, JR (2003) Comparison of beta-lactamases of classes A and D: 1.5-A crystallographic structure of the class D OXA-1 oxacillinase. Protein Sci 12: 82–91 12493831 10.1110/ps.0224303 PMC2312410

[103] Golemi, D, Maveyraud, L, Vakulenko, S, Samama, JP, Mobashery, S (2001) Critical involvement of a carbamylated lysine in catalytic function of class D beta-lactamases. Proc Natl Acad Sci USA 98: 14280–14285 11724923 10.1073/pnas.241442898 PMC64673

[104] Garcia-Viloca, M, Poulsen, TD, Truhlar, DG, Gao, J (2004) Sensitivity of molecular dynamics simulations to the choice of the X-ray structure used to model an enzymatic reaction. Protein Sci 13: 2341–2354 15322278 10.1110/ps.03504104 PMC2280009

[105] Schnoes, AM, Brown, SD, Dodevski, I, Babbitt, PC (2009) Annotation error in public databases: misannotation of molecular function in enzyme superfamilies. PLoS Computational Biology 5: e1000605 20011109 10.1371/journal.pcbi.1000605 PMC2781113

